# International Opinions on Grading of Urothelial Carcinoma: A Survey Among European Association of Urology and International Society of Urological Pathology Members

**DOI:** 10.1016/j.euros.2023.03.019

**Published:** 2023-05-10

**Authors:** Irene J. Beijert, Liang Cheng, Fredrik Liedberg, Karin Plass, Sean R. Williamson, Paolo Gontero, Maria J. Ribal, Marko Babjuk, Peter C. Black, Ashish M. Kamat, Ferran Algaba, David M. Berman, Arndt Hartmann, Alexandra Masson-Lecomte, Morgan Rouprêt, Antonio Lopez-Beltran, Hemamali Samaratunga, Shahrokh F. Shariat, A. Hugh Mostafid, Murali Varma, Steven Shen, Maximilian Burger, Toyonori Tsuzuki, Joan Palou, Eva M. Compérat, Richard J. Sylvester, Theo H. van der Kwast, Bas W.G. van Rhijn, Michelle R. Downes

**Affiliations:** a Department of Surgical Oncology (Urology), Netherlands Cancer Institute – Antoni van Leeuwenhoek Hospital, Amsterdam, The Netherlands; b Department of Urology, Amsterdam University Medical Centers, Vrije Universiteit, Amsterdam, The Netherlands; c Department of Pathology and Laboratory Medicine, Brown University Warren Alpert Medical School, Lifespan Academic Medical Center and Legorreta Cancer Center at Brown University, Providence, RI, USA; d Department of Translational Medicine, Lund University, Malmö, Sweden; e Department of Urology, Skåne University Hospital, Malmö, Sweden; f European Association of Urology, Non-Muscle Invasive Bladder Cancer Guidelines Panel, Arnhem, The Netherlands; g European Association of Urology, Guidelines Office Board, Arnhem, The Netherlands; h Department of Pathology, Cleveland Clinic, Cleveland, OH, USA; i Department of Urology, Città della Salute e della Scienza, University of Torino School of Medicine, Torino, Italy; j Department of Urology, Hospital Clínic de Barcelona, Universitat de Barcelona, Barcelona, Spain; k Department of Urology, Teaching Hospital Motol and 2nd Faculty of Medicine, Charles University Praha, Prague, Czech Republic; l Department of Urology, Comprehensive Cancer Center, Medical University Vienna, Vienna General Hospital, Vienna, Austria; m Department of Urologic Sciences, University of British Columbia, Vancouver, BC, Canada; n Department of Urology, University of Texas, MD Anderson Cancer Center, Houston, TX, USA; o Department of Pathology, Fundació Puigvert, Universitat Autónoma de Barcelona, Barcelona, Spain; p Kingston Health Sciences Centre, Queen’s University, Kingston, ON, Canada; q Department of Pathology and Molecular Medicine, Queen’s University, Kingston, ON, Canada; r Institute of Pathology, Friedrich-Alexander-University of Erlangen-Nürnberg, Erlangen, Germany; s Department of Urology, Université de Paris, APHP, Saint Louis Hospital, Paris, France; t Department of Urology, Pitié Salpétrière Hospital, AP-HP, GRC n°5, ONCOTYPE-URO, Sorbonne University, Paris, France; u Department of Morphological Sciences, University of Cordoba Medical School, Cordoba, Spain; v Anatomic Pathology, Champalimaud Clinical Center, Lisbon, Portugal; w Department of Pathology, Aquesta Uropathology and University of Queensland, Brisbane, Australia; x Department of Urology, The Stokes Centre for Urology, Royal Surrey Hospital, Guildford, UK; y Department of Cellular Pathology, University Hospital of Wales, Cardiff, UK; z Department of Pathology Genomic Medicine, Houston Methodist Hospital and Weill Cornell Medical College, Houston, TX, USA; aa Department of Urology, Caritas St. Josef Medical Center, University of Regensburg, Regensburg, Germany; ab Department of Surgical Pathology, Aichi Medical University Hospital, Nagakute, Japan; ac Department of Urology, Fundacio Puigvert, Universitat Autònoma de Barcelona, Barcelona, Spain; ad Department of Pathology, Medical University Vienna, Vienna General Hospital, Vienna, Austria; ae Laboratory Medicine Program, University Health Network, Princess Margaret Cancer Center, University of Toronto, Toronto, Canada; af Division of Anatomic Pathology, Precision Diagnostics and Therapeutics Program, Sunnybrook Health Sciences Centre, Toronto, ON, Canada; ag Department of Laboratory Medicine and Pathobiology, University of Toronto, Toronto, ON, Canada

**Keywords:** Bladder, Cancer, Grading, Survey, European Association of Urology, International Society of Urological Pathology, WHO1973, WHO2004

## Abstract

**Background:**

Grade of non–muscle-invasive bladder cancer (NMIBC) is an important prognostic factor for progression. Currently, two World Health Organization (WHO) classification systems (WHO1973, categories: grade 1–3, and WHO2004 categories: papillary urothelial neoplasm of low malignant potential [PUNLMP], low-grade [LG], high-grade [HG] carcinoma) are used.

**Objective:**

To ask the European Association of Urology (EAU) and International Society of Urological Pathology (ISUP) members regarding their current practice and preferences of grading systems.

**Design, setting, and participants:**

A web-based, anonymous questionnaire with ten questions on grading of NMIBC was created. The members of EAU and ISUP were invited to complete an online survey by the end of 2021. Thirteen experts had previously answered the same questions.

**Outcome measurements and statistical analysis:**

The submitted answers from 214 ISUP members, 191 EAU members, and 13 experts were analyzed.

**Results and limitations:**

Currently, 53% use only the WHO2004 system and 40% use both systems. According to most respondents, PUNLMP is a rare diagnosis with management similar to Ta-LG carcinoma. The majority (72%) would consider reverting back to WHO1973 if grading criteria were more detailed. Separate reporting of WHO1973-G3 within WHO2004-HG would influence clinical decisions for Ta and/or T1 tumors according the majority (55%). Most respondents preferred a two-tier (41%) or a three-tier (41%) grading system. The current WHO2004 grading system is supported by a minority (20%), whereas nearly half (48%) supported a hybrid three- or four-tier grading system composed of both WHO1973 and WHO2004. The survey results of the experts were comparable with ISUP and EAU respondents.

**Conclusions:**

Both the WHO1973 and the WHO2004 grading system are still widely used. Even though opinions on the future of bladder cancer grading were strongly divided, there was limited support for WHO1973 and WHO2004 in their current formats, while the hybrid (three-tier) grading system with LG, HG-G2, and HG-G3 as categories could be considered the most promising alternative.

**Patient summary:**

Grading of non–muscle-invasive bladder cancer (NMIBC) is a matter of ongoing debate and lacks international consensus. We surveyed urologists and pathologists of European Association of Urology and International Society of Urological Pathology on their preferences regarding NMIBC grading to generate a multidisciplinary dialogue. Both the “old” World Health Organization (WHO) 1973 and the “new” WHO2004 grading schemes are still used widely. However, continuation of both the WHO1973 and the WHO2004 system showed limited support, while a hybrid grading system composed of both the WHO1973 and the WHO2004 classification system may be considered a promising alternative.

## Take Home Message

Both the World Health Organization (WHO) 1973 and WHO2004 grading systems are used widely. However, there was limited support for WHO1973 and WHO2004 in their current formats, while the hybrid (three-tier) grading system with low-grade, high-grade (HG)-G2, and HG-G3 as categories could be considered the most promising alternatives.

## 1 Introduction

As non–muscle-invasive bladder cancer (NMIBC) is a heterogeneous disease, accurate risk stratification, based on both clinical and pathological factors, is crucial to determine optimal treatment and surveillance strategies for each patient. Histological grade of NMIBC is an important prognostic factor for progression to muscle-invasive and/or metastatic disease [1], [2].

**Fig. 1:**
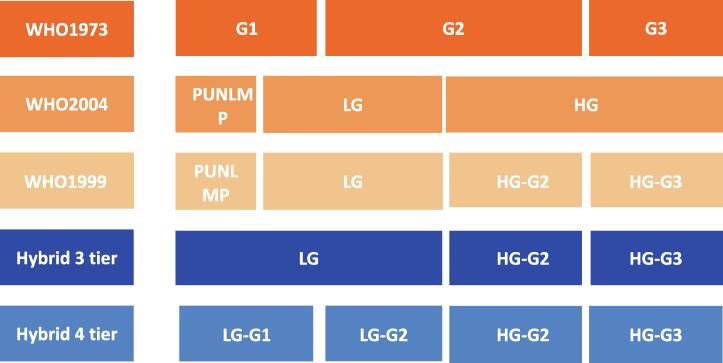
Overview of bladder cancer grading systems. Graphical comparison of different bladder cancer grading systems and the cutoff between grades. G = grade; HG = high grade; LG = low grade; PUNLMP = papillary urothelial neoplasm of low malignant potential; WHO = World Health Organization.

The World Health Organization (WHO) adopted the first bladder cancer grading classification in 1973 dividing papillary urothelial carcinomas into grades 1–3 (G1, G2, and G3) [3]. The lack of clear histological criteria for the three WHO1973 grades with the majority of carcinomas being classified in the middle group (G2) prompted the proposal of a new grading classification scheme in 1998 [3], [4]. This WHO/International Society of Urological Pathology (ISUP) scheme consisted of papillary urothelial neoplasm of low malignant potential (PUNLMP), and low-grade (LG) and high-grade (HG) noninvasive papillary urothelial carcinoma with more detailed histological criteria [4]. The PUNLMP entity was created to avoid the ‘’cancer’’ label in a category of patients with a presumed low risk of recurrence [4]. Subsequently, the WHO/ISUP1998 classification was modified into a four-tier classification (WHO1999), in which HG was subclassified in G2 and G3 with more resemblance to the older WHO1973 grading system [5], [6]. Ultimately, the WHO adopted the three-tier 1998 classification in 2004 and subsequently in 2016 [7] and 2022 (Fig. 1) [8].

Unlike the WHO1973 system, the WHO2004 grading system was adopted on the basis of its clear histological definitions of each category but without clinical evidence supporting its prognostic value [6]. Eventually, Soukup et al. [9] compared the prognostic performance of WHO1973 and WHO2004 in a systematic review in 2017 and concluded that WHO2004 did not outperform WHO1973, yet suggested that larger studies with individual patient data (IPD) were needed since the review was based on a small number of clinical studies with relatively few patients (five studies with in total 931 patients for recurrence; seven studies with in total 1371 patients for progression). Therefore, van Rhijn et al. [10] collected IPD of 5145 patients to compare both grading systems and showed that the prognostic value of WHO1973 for predicting progression of primary Ta-T1 NMIBC was better than that of WHO2004 in a real-world setting. Moreover, a combination of both systems (LG/G1, LG/G2, HG/G2, and HG/G3; hybrid four tier) proved superior to either system alone (Fig. 1) [10]. Their results did not support PUNLMP as a separate grade category because the prognosis was comparable with Ta-LG and the diagnosis had become extremely rare [11]. Subsequently, the ISUP established a multidisciplinary workgroup of nine pathologists and four urologists with long-standing expertise in the field to determine the requirements for an optimal classification system based on a literature review of clinical and molecular evidence. These 13 experts were also surveyed regarding several aspects of the WHO1973 and WHO2004 grading systems [12].

The aim of this current EAU-ISUP survey, composed of the same questions as for the multidisciplinary workgroup [12], was to ask a larger sample of urologists (EAU) and pathologists (ISUP) about their preferences regarding NMIBC grade to generate a multidisciplinary dialogue. This survey, in part, also informed a subsequent ISUP consensus bladder conference meeting in September 2022, the proceedings of which will be published in a separate manuscript. The purpose of this survey was not to form guidelines or recommendations, as those should primarily be based on clinical evidence.

## 2 Patients and methods

With permission of the ISUP and the EAU central guidelines office, a web-based anonymous questionnaire was launched with ten questions on grading of NMIBC. All questions were multiple-choice type, and some questions had the option to provide comments, if the preferred option was not available. The invitation to participate in the survey was circulated by e-mails to the EAU and ISUP members by the end of 2021. Survey questions were similar to the survey questions in the study by van der Kwast et al. [12] and are displayed in the Supplementary material. The answers of the surveyed experts were obtained as well [12]. Descriptive statistics were summarized as frequencies and percentages.

Overall, 418 (ie, 214 ISUP, 191 EAU, and 13 expert) survey responses were obtained. The ISUP member respondents consisted of 176 pathologists and 38 pathologists in training. The EAU respondents comprised 175 urologists, seven pathologists, and nine scientists/PhD candidates or other. Additional demographics (eg, workplace and region) are shown in Table 1.

**Table 1:** Demographics of survey respondents

Member	ISUP	EAU	Expert
Profession, *n* (%)			
Urologist	–	175 (92)	4 (31)
Pathologist	214 (100)	7 (4)	9 (69)
Scientist/PhD candidate	–	5 (2)	–
Other	–	4 (2)	–
Workplace, *n* (%)			
Academic medical center/medical school–affiliated hospital	132 (62)	120 (63)	12 (92)
Community practice hospital	50 (23)	56 (29)	–
Commercial third party hospital	23 (11)	8 (4)	1 (8)
Other	9 (4)	7 (4)	–
Geographical location, *n* (%)^a^			
Europe	84 (39)	–	6 (46)
North America	64 (30)	–	6 (46)
Central and South America (including Mexico)	21 (10)	–	–
Australia and Asia	19 (9)	–	1 (8)
Middle East/Africa	6 (3)	–	–
Other	20 (9)	–	–
Pathology practice experience (yr), *n* (%)^a^			
≥11	139 (65)	–	–
5–10	43 (20)	–	–
<5	32 (15)	–	–

EAU = European Association of Urology; ISUP = International Society of Urological Pathology.

a The geographical distribution and work experience of EAU members was not obtained.

## 3 Results

### 3.1 Currently utilized grading system

When reporting a papillary noninvasive urothelial carcinoma, most (220/418, 53%) use the WHO2004 grading system followed by 169/418 (40%) who report both the WHO1973 and the WHO2004 grading system. A minority (17/418, 4%) uses only the WHO1973 grading system*.*

**Fig. 2:**
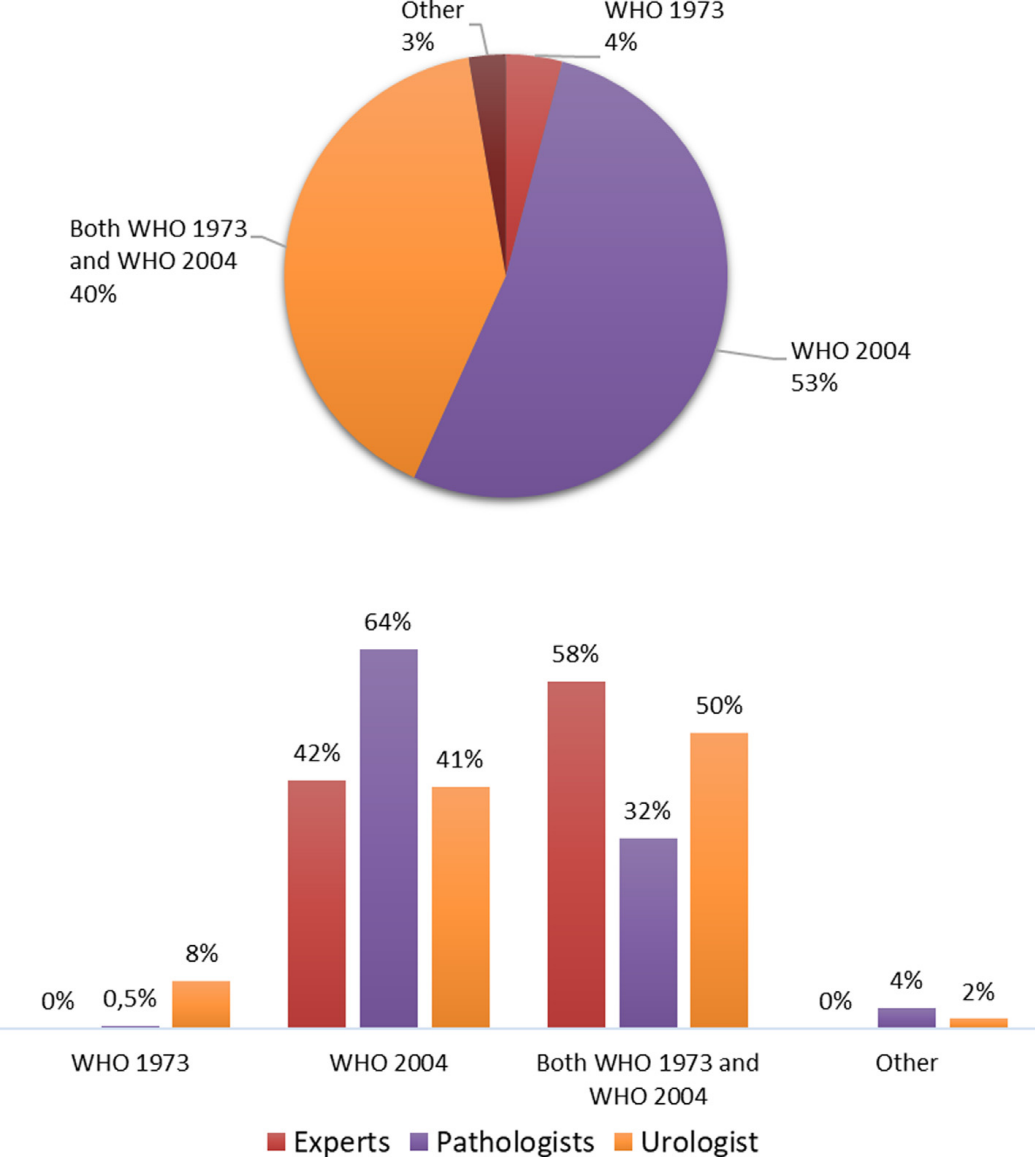
Which grading system do you currently use when reporting a papillary noninvasive urothelial carcinoma? This question was answered by 418 respondents (pie chart), of whom 13 were experts, 221 pathologists (214 ISUP and seven EAU), and 175 urologists (bar charts). Of note, the grading system utilized by urologists is likely a reflection of the grading system used by their pathologist(s). EAU = European Association of Urology; ISUP = International Society of Urological Pathology; WHO = World Health Organization.

The majority of pathologists (141/221, 64%), but not urologists (71/175, 41%) and experts (5/13, 42%), use only the WHO2004 grading system. The combination of WHO1973 and WHO2004 grading is used by half (87/175, 50%) of urologists, a third (71/221, 32%) of pathologists, and more than half (7/13, 58%) of experts. The WHO1973 grading system is used alone in the daily practice of a minority (8% of urologists, 0.5% of pathologists, and no experts; Fig. 2). Of note, the utilized grading system by urologists is likely a reflection of the grading system used by “their” pathologist(s).

Most (59/64, 92%) of the pathologists from North America use only the WHO2004 grading system, while the majority (50/84, 60%) of the pathologists from Europe use both WHO1973 and WHO2004, followed by 32/84 (38%) who use only WHO2004. Four out of six (67%) experts from North America use the WHO2004, and the other two use both WHO1973 and WHO2004. All five experts from Europe use both WHO1973 and WHO2004.

### 3.2 Opinions on PUNLMP

**Fig. 3:**
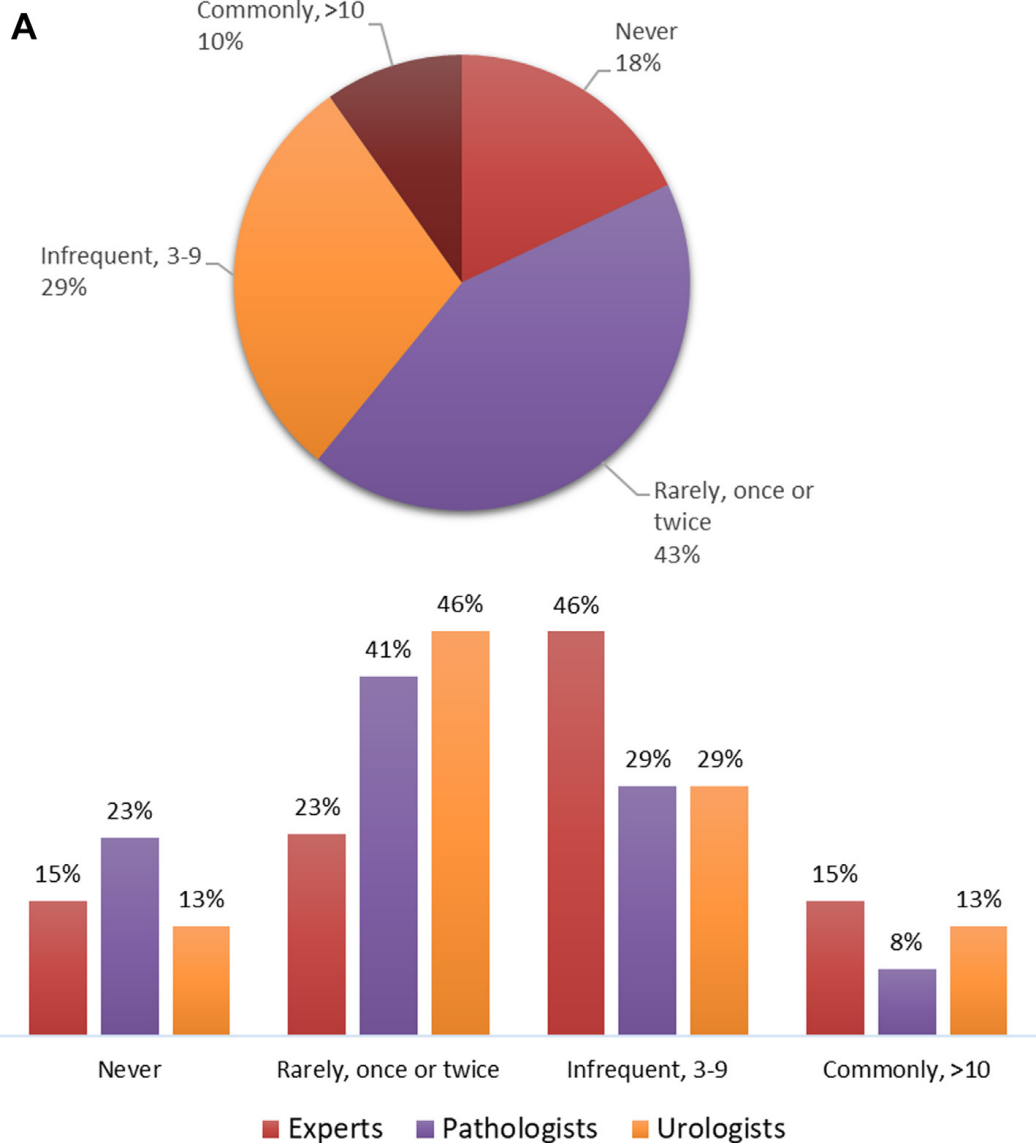

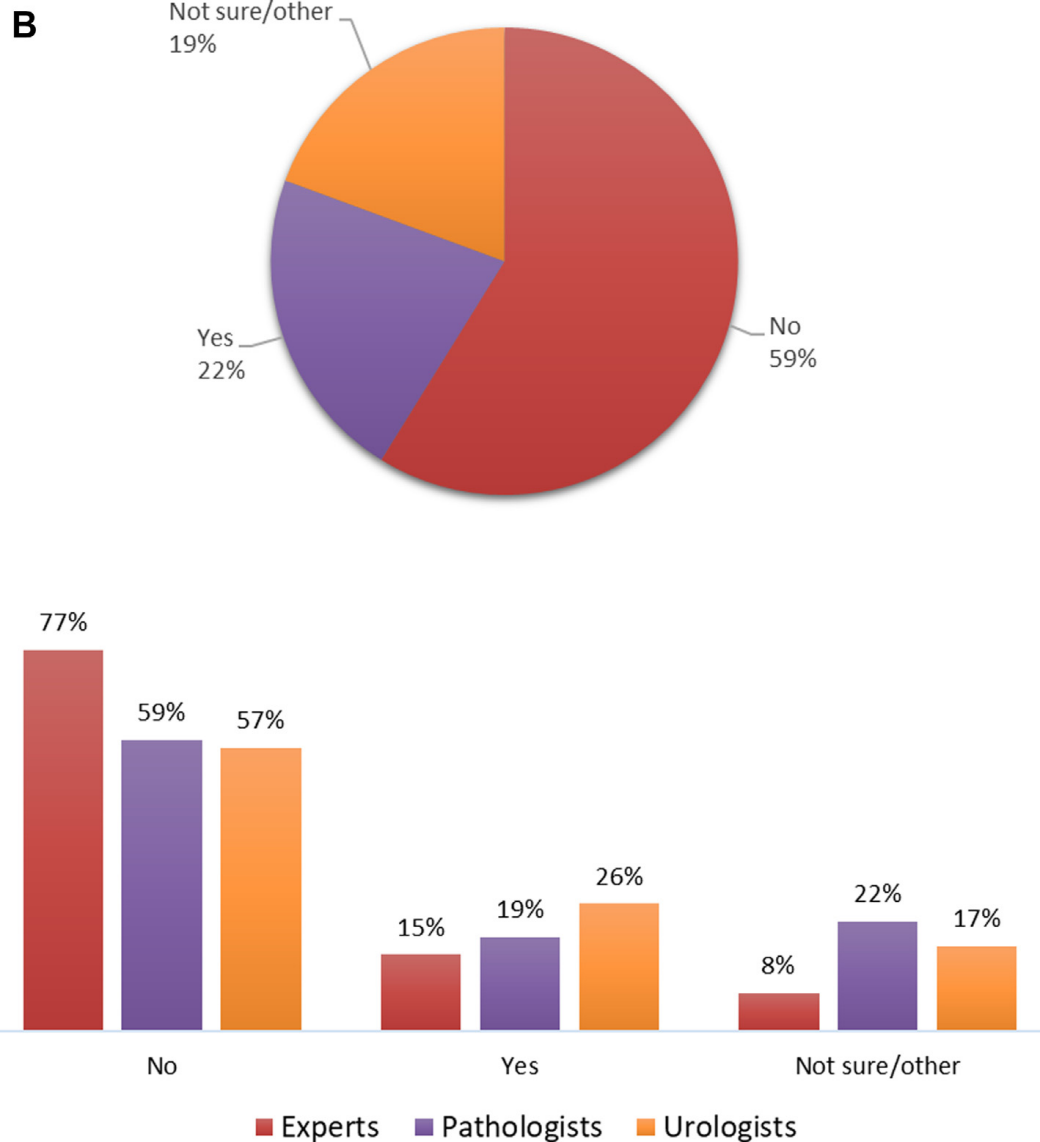
(A) How many cases of PUNLMP did you encounter in 2020? This question was answered by 417 respondents (pie chart), of whom 13 were experts, 221 pathologists (214 ISUP and seven EAU), and 175 urologists (bar charts). (B) Do you think treatment/management of PUNLMP should be different from Ta low grade urothelial carcinoma? This question was answered by 418 respondents (pie chart), of whom 13 were experts, 221 pathologists (214 ISUP and seven EAU), and 175 urologists (bar charts). EAU = European Association of Urology; ISUP = International Society of Urological Pathology; PUNLMP = papillary urothelial neoplasm of low malignant potential.

In 2020, 193/418 (46%) respondents encountered more than 50 Ta urothelial carcinomas (excluding PUNLMP), 129/418 (31%) encountered 20–50, cases and 93/418 (23%) encountered <20 cases. In the same year, 75/417 (18%) never encountered or diagnosed PUNLMP, 179/417 (43%) rarely (once or twice) saw a PUNLMP case, 122/417 (29%) did so infrequently (three to nine times), and 41/418 (10%) commonly (more than ten times) saw a PUNLMP case. Responses between urologists and pathologists were similar, though pathologists seem to diagnose fewer PUNLMP cases than urologists encounter (Fig. 3A). Moreover, pathologists working in Europe diagnose PUNLMP less frequently (62/84, 74% never or rarely) than their North American colleagues (35/64, 54% never or rarely).

Management of PUNLMP should be similar to that of Ta-LG urothelial carcinoma according to the majority (246/418, 59%), with similar responses between urologists and pathologists. Urologists, pathologists, and experts who think that treatment of PUNLMP should be different from Ta-LG are the minority (26% [45/175], 19% [42/221], and 15% [2/13], respectively; Fig. 3B).

### 3.3 Opinions on WHO1973

**Fig. 4:**
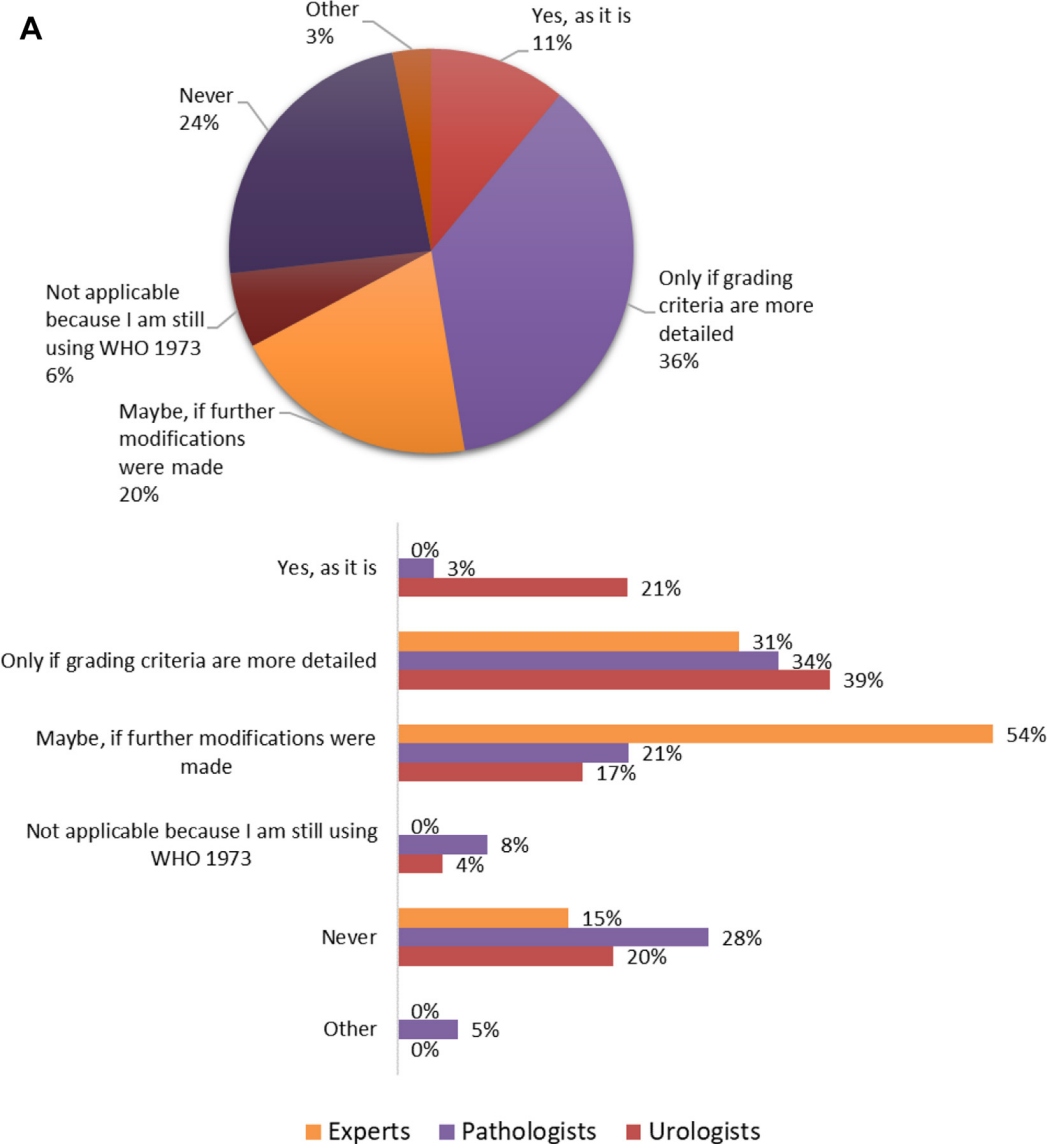

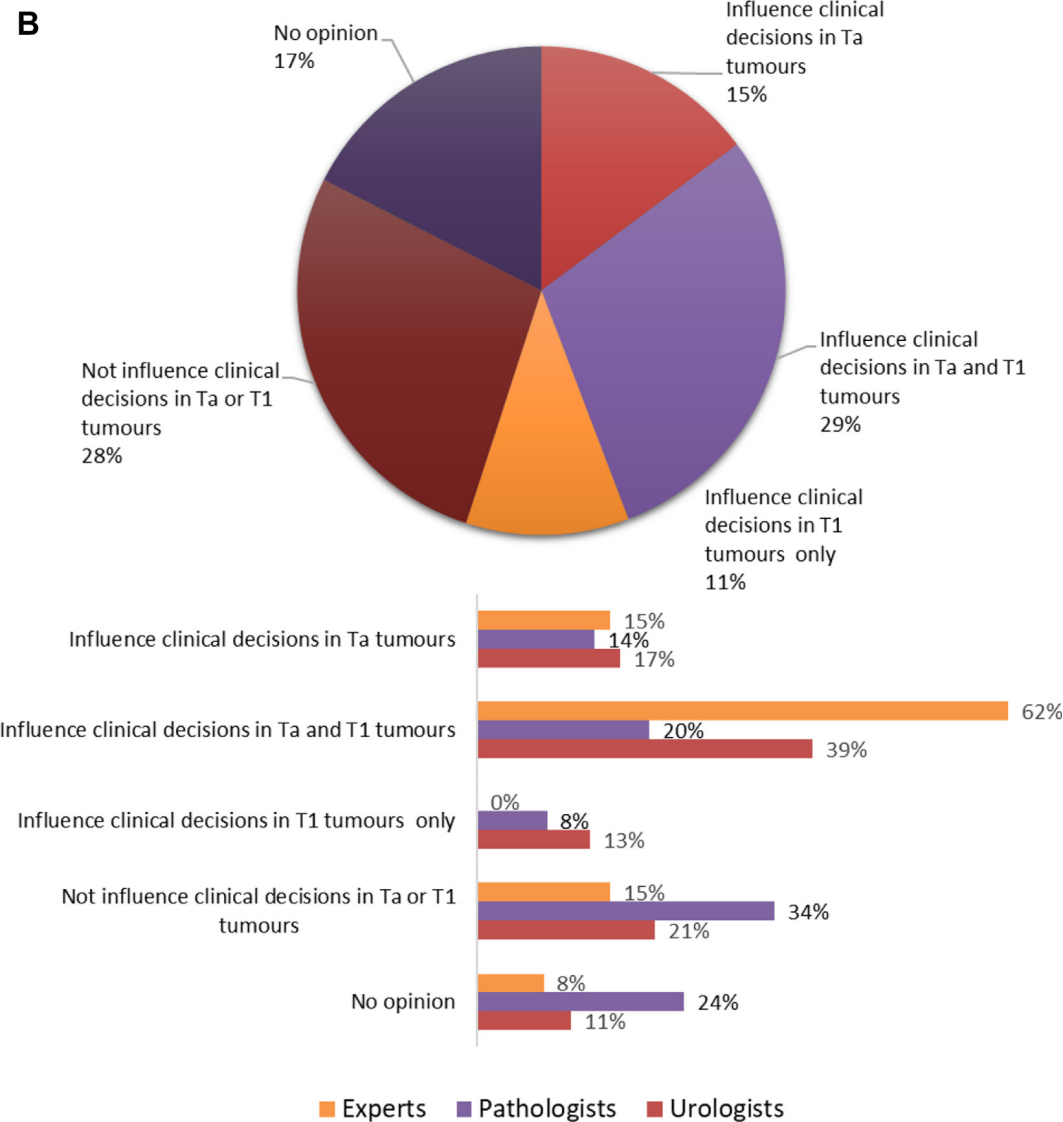
(A) Would you consider reverting back to the WHO1973 grading system? This question was answered by 417 respondents (pie chart), of whom 13 were experts, 221 pathologists (214 ISUP and seven EAU), and 174 urologists (bar charts). (B) With regard to WHO1973 grade 3, do you think its distinction from WHO2004 high grade would influence clinical management? This question was answered by 418 respondents (pie chart), of whom 13 were experts, 221 pathologists (214 ISUP and seven EAU), and 175 urologists (bar charts). EAU = European Association of Urology; ISUP = International Society of Urological Pathology; T = stage; WHO = World Health Organization.

Reverting back to the WHO1973 grading system in the current form (including respondents still using WHO1973) would be an option for 71/417 (17%), including 25% of urologists, 11% pathologists, and no experts. More than half of urologists (97/174, 56%) and pathologists (122/221, 55%) and most experts (11/13, 85%) would consider using WHO1973, provided that further modifications were made or grading criteria were more detailed (Fig. 4A). Reverting back to WHO1973 would never be an option for 99/417 (24%) of respondents. Furthermore, 145/220 (66%) of the respondents who currently use only WHO2004 would consider to revert back to WHO1973 as it is (18/220, 8%) or provided that further modifications are made (127/220, 58%).

Dual reporting of WHO1973-G3 along with WHO2004-HG would influence clinical decisions for Ta and/or T1 tumors according to the majority (230/418, 55%). More urologists (120/175, 69%) and experts (10/13, 77%) than pathologists (92/221, 42%) believe that separate reporting of WHO1973-G3 next to WHO2004-HG would influence clinical decisions for Ta and/or T1 tumors. Notably, a quarter (53/221, 24%) of pathologists are uncertain on this subject (Fig. 4B).

### 3.4 Preferences regarding (future) optimal grading systems

**Fig. 5:**
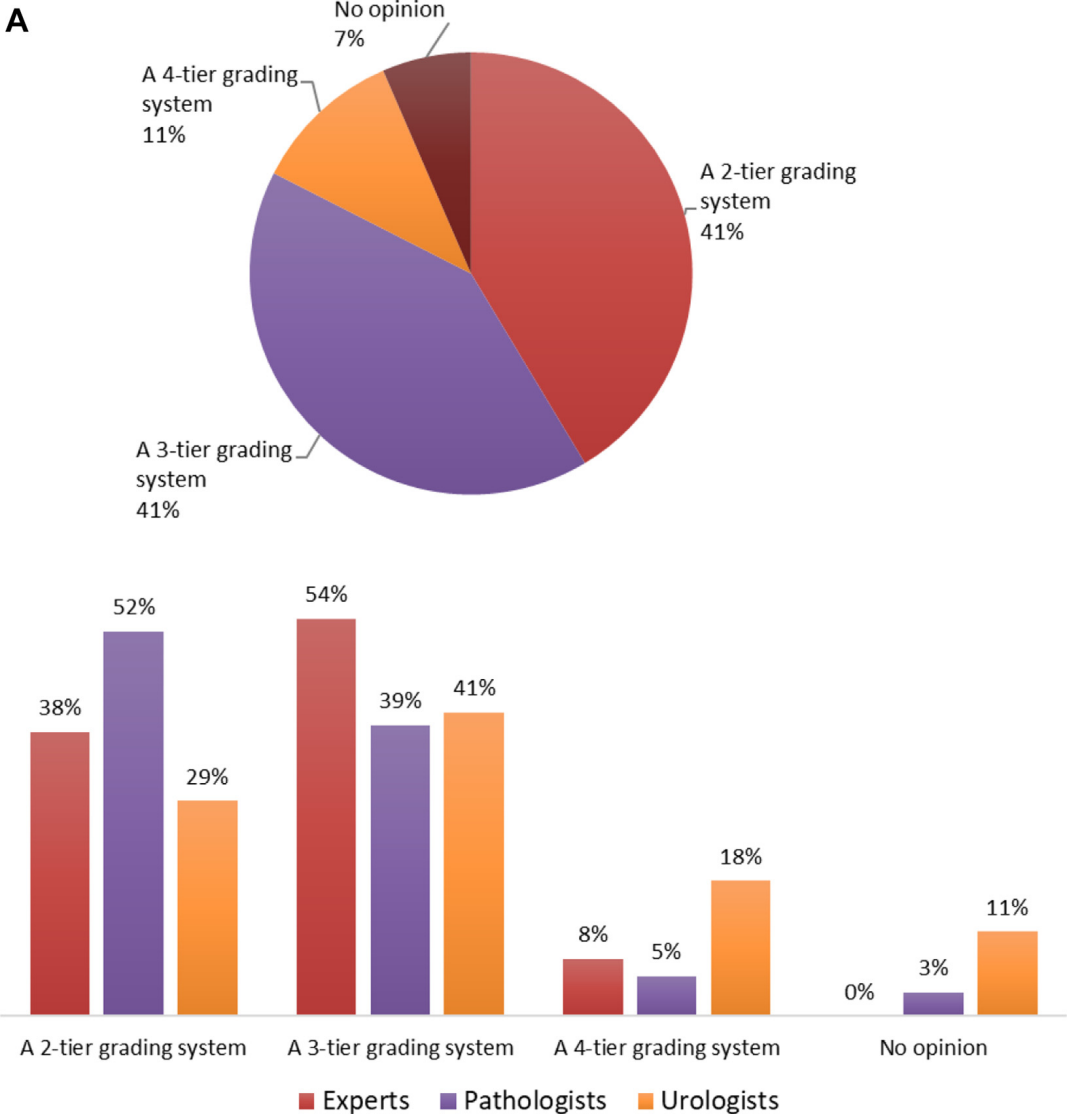

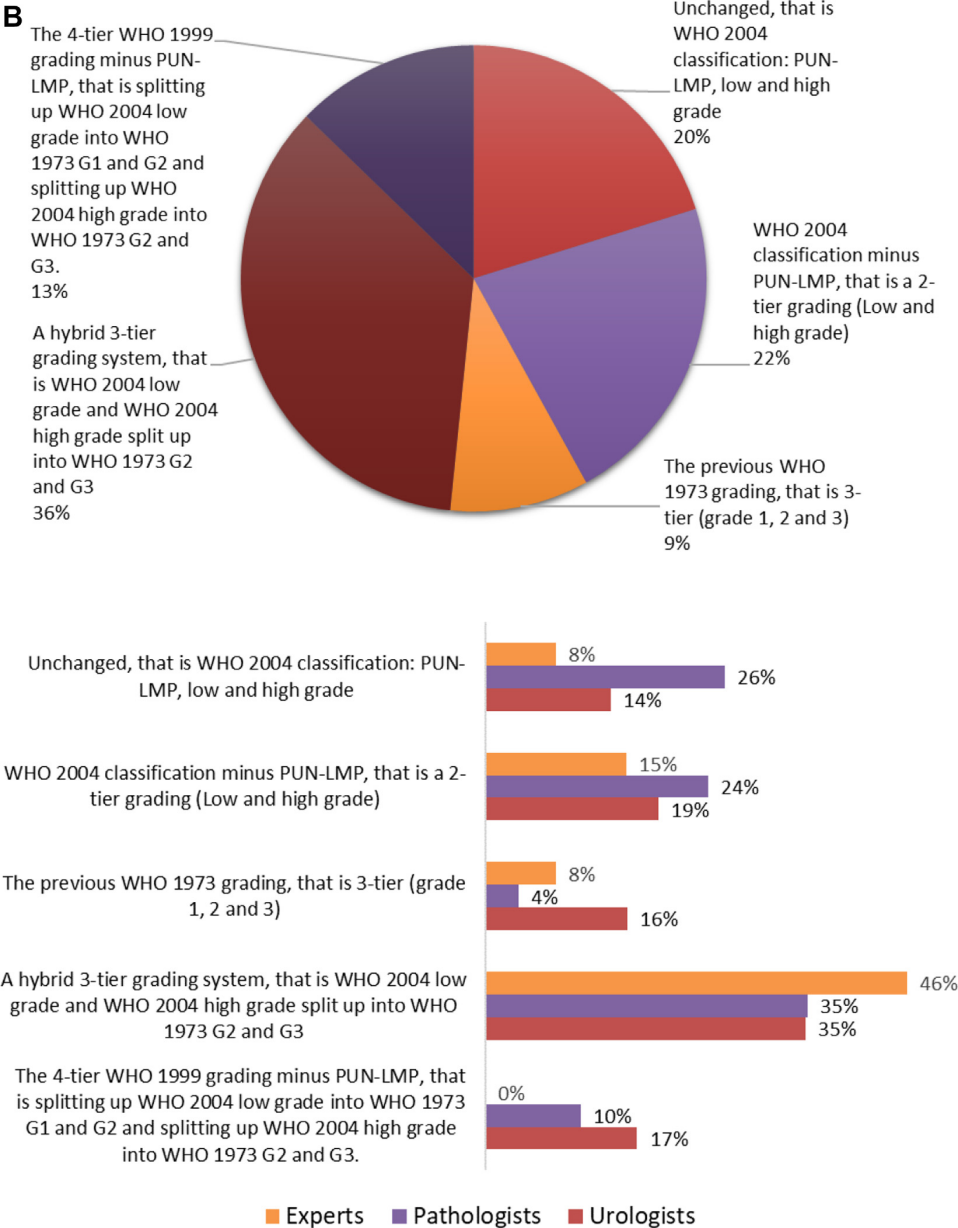
(A) What do you prefer for bladder cancer grading? This question was answered by 418 respondents (pie chart), of whom 13 were experts, 221 pathologists (214 ISUP and seven EAU), and 175 urologists (bar charts). (B) Do you think that What should a future grading system be? This question was answered by 414 respondents (pie chart), of whom ten were experts, 221 pathologists (214 ISUP and seven EAU), and 174 urologists (bar charts). EAU = European Association of Urology; ISUP = International Society of Urological Pathology; T = stage; WHO = World Health Organization.

Similar numbers of survey respondents prefer a two-tier (173/418, 41%) or a three-tier (172/418, 41%) grading system. More than half of the pathologists (115/221, 52%) and a third of the urologists (51/175, 29%) prefer a two-tier grading system, and similar percentages (urologists 39% and pathologists 41%) prefer a three-tier grading system. Most experts prefer a three-tier grading system (7/13, 54%), followed by a two-tier grading system (5/13, 38%). A minority (46/418, 11%) prefers a four-tier grading system with more urologists (32/175, 18%) than pathologists (12/221, 5%) or experts (1/13, 8%; Fig. 5A).

With respect to the question of what a future grading system should look like, a minority (20%, including 14% urologists, 26% pathologists, and 8% experts) would like to keep WHO2004 as it is, that is, PUNLMP, LG, HG. This opinion is shared by more North American pathologists (31%) than European pathologists (18%). In total, 174/414 (42%) respondents would continue with the WHO2004 grading classification. However, more than half (90/174, 52%) would omit PUNLMP from WHO2004 and turn it into a two-tier grading system (LG and HG). Among urologists, there was limited support for using an unmodified WHO1973 grading system (27/174, 16%) or the current WHO2004 grading system without modification (24/174, 14%). Very few pathologists (8/221, 4%) would prefer the previous WHO1973 grading system as it currently exists (Fig. 5B).

A hybrid three-tier grading system, that is WHO2004-LG and splitting up WHO2004-HG into WHO1973-G2 and WHO1973-G3, is preferred by 61/174 (35%) urologists, 78/221 (35%) pathologists, and 6/13 (46%) experts. A hybrid four-tier grading system, that is, WHO2004 minus PUNLMP and splitting up WHO2004-LG into G1 and G2 and WHO2004-HG into G2 and G3, would be the optimal system for 29/174 (17%) urologists, 23/221 (10%) pathologists, and no experts (Fig. 5B). Overall, a hybrid three- or four-tier grading system would be preferred by 48%, including 90/174 (52%) urologists, 101/221 (46%) pathologists, and 6/13 (46%) experts.

## 4 Discussion

Histological grade of NMIBC is an important prognostic factor for progression to muscle-invasive and/or metastatic disease [1], [2]. However, grading of NMIBC is a matter of continuing debate as it remains questionable whether the WHO2004 classification system actually improved risk stratification for clinical treatment and surveillance strategies [9], [10], [11], [13]. The aim of this EAU-ISUP survey was to ask a large sample of urologists and pathologists from the EAU and ISUP about their preferences regarding grading NMIBC to generate a multidisciplinary dialogue on this contentious issue.

The most commonly utilized grading systems worldwide are the WHO1973 and WHO2004 classification systems. However, there is no international agreement on their use and the two classification systems are not directly translatable into each other due to different cutoff points between grades. The American Urological Association and the WHO recommend using the WHO2004 grading system, whereas the EAU supports the use of both grading systems [1], [14], [15]. Moreover, the ISUP recently suggested a hybrid three-tier (LG, HG/G2, and HG/G3) grading classification [12]. In this survey, we found marked variability in the currently utilized grading schemes as 53% use WHO2004 and 44% still use WHO1973 (together with WHO2004). The variability is partly dependent on geographical location as the majority—though not all—of pathologists from Europe (60%) use both WHO1973 and WHO2004, and most pathologists from North America (92%) use the WHO2004 grading system. Moreover, approximately half of the urologists—all EAU respondents—currently use both the WHO1973 and the WHO2004 grading system. Hence, there is no international consensus on NMIBC grading.

The PUNLMP category has been subject to debate since its introduction. Comparable recurrence and progression rates between PUNLMP and Ta-LG carcinomas were recently confirmed by Hentschel et al. [11]. In addition, a strong decline in PUNLMP diagnosis with a decrease from 31% before 2000 to only 1% after 2010, has been observed [11]. Therefore, Hentschel et al. [11] concluded that there was limited support to retain PUNLMP as a separate grade category. Nevertheless, from population-based data in the context of WHO1999, patients with primary LG/G1 tumors were reported to have an increased risk of recurrence compared with those with PUNLMP [16]. The controversy about this category was confirmed by our survey results, since 61% of respondents encounter a PUNLMP lesion never or rarely in daily clinical practice and barely 10% does so commonly. Moreover, nearly 60% of respondents believe that the management of PUNLMP should be similar to Ta-LG carcinomas. In addition, from the respondents who preferred to continue the WHO2004 grading classification in the future (*n* = 174), more than half would omit PUNLMP, thereby turning the WHO2004 grading system into a two-tier grading system consisting of only LG and HG. Taken together, the use of PUNLMP as a separate category within WHO2004 is also questioned by the current survey results.

The major criticism of the WHO1973 grading system has always been the lack of clearly defined criteria, particularly for grade 2 cancers (“not grade 1, not grade 3”), which consequently led to a ‘’default’’ G2 diagnosis of a large clinically heterogeneous bladder cancer population. Nevertheless, it remains questionable whether the implementation of the WHO2004 classification solved the interobserver variability issues and actually improved NMIBC grading [9]. Even though the adoption of WHO2004 was meant to reduce interobserver variability by defining clear histological criteria, subsequent studies on the reproducibility of WHO2004 showed that observer variability did not really improve by formulating these detailed criteria for each grade category [9], [13]. A recent study by van Rhijn et al. [10] compared both grading systems and showed that the prognostic value of WHO1973 for predicting progression of primary Ta-T1 NMIBC was better than that of WHO2004. Although WHO1973 may seem outdated, 44% of our survey respondents still use WHO1973 in daily clinical practice, mostly in the context of dual grading with WHO2004. Further, reporting of WHO1973-G3 subgroup in WHO2004-HG carcinomas would have a clinical impact according to 69% of urologists and 77% of experts. Somewhat surprisingly, reverting back to WHO1973 would be an option for 73% of the survey respondents, yet for most (56%) only if further modifications are made to the system or grading criteria are more detailed.

Strikingly, retention of the current iteration of the WHO2004 grading system is favored by only 20% of survey respondents, and a small number (10%) of respondents would favor the WHO1973 classification in its current form. Approximately half the respondents would prefer hybrid three- or four-tier grading classification based on both WHO1973 and WHO2004, with the majority favoring three-tier grading, that is, WHO2004 minus PUNLMP and splitting up WHO2004-HG into WHO1973-G2 and WHO1973-G3. Similar results were found in the survey conducted among 13 ISUP experts, as only one would prefer WHO2004 as it is and most (*n* = 6, 46%) preferred the three-tier hybrid grading system option [12]. From a clinical point of view, van Rhijn et al. [10] showed in 5145 patients from 17 hospitals that a combination of both WHO1973 and WHO2004 (LG/G1, LG/G2, HG/G2, and HG/G3) was superior to either system alone [10]. Another contemporary study by Downes et al. [17] in 609 patients from two North American hospitals also reported that the hybrid three- or four-tier grading system is a better prognosticator for progression than either WHO1973 or WHO2004 alone. Both studies strongly support the separation of the WHO2004-HG category into WHO1973-G2 and WHO1973-G3 with clinically relevant differences in progression (van Rhijn et al. [10] 8% vs 19%; Downes et al. [17] 27% vs 44%). In contrast, the separation of WHO2004-LG into WHO1973-G1 and WHO1973-G2 was found to be clinically less relevant because of the lower progression risks (van Rhijn et al. [10] 1% vs 4%; Downes et al. [17] 3% vs 4%). Likewise, an ISUP multidisciplinary workgroup on NMIBC grade recently suggested to subdivide the heterogeneous WHO2004-HG category into WHO1973-G2 and WHO1973-G3, based on both clinical and molecular evidence [12]. Based on our survey results, opinions on a future grading system are quite scattered. Nonetheless, there was clearly limited support for the continuation of both WHO1973 and WHO2004 in their current form while a hybrid (three- or four-tier) grading system received more support. Therefore, a hybrid grading system could indeed be a good option for BC grading in the future as it is also supported by clinical data [10], [17] and previous suggestions to introduce such a system (WHO 1999) [5].

One of the limitations of our survey is that the number respondents represent a relatively small sample of all EAU and ISUP members. Another limitation is that all urologists are EAU members, and therefore the results predominantly reflect European urology practice. Nevertheless, since experts in the field, and both urologists and pathologists working in various parts of the world and in different hospital settings have responded, our results may be a reasonable reflection of reality. Obviously, the aim of this survey was not to establish guidelines or recommendations, as those should primarily be based on clinical evidence. We believe that our survey proved useful to evaluate the discordance/concordance between physicians (urologists/pathologists) when assessing the impact of grade on guidelines and in current, as well as, future daily clinical practice.

## 5 Conclusions

Grading of NMIBC is a matter of ongoing debate, and there seems to be no international consensus. The “old” WHO1973 system is still widely used in some form and in some jurisdictions. The present survey results among experts and members of the ISUP and the EAU showed that PUNLMP as a separate category was not used widely apart from by a minority in North America. Strikingly, reverting back to WHO1973 grading would be an option for the majority of pathologists and urologists, provided that grading criteria are more detailed in the future. The vast majority of the respondents believed that subdivision of the HG category of WHO2004 by WHO1973-G2 and WHO1973-G3 would influence clinical management of NMIBC. There seems to be only limited support among survey participants for the continuation of the existing WHO2004 (20%) and WHO1973 (10%) grading classification systems in their current form. Our survey and recent clinical data showed that a hybrid, preferably three-tier (based on both WHO1973 and WHO2004 with the categories LG, HG-G2, and HG-G3), grading system seems to be a promising, clinically prognostic alternative for the future.

  ***Author contributions:*** Irene J. Beijert had full access to all the data in the study and takes responsibility for the integrity of the data and the accuracy of the data analysis.

*Study concept and design*: Beijert, Cheng, Williamson, Plass, Liedberg, Babjuk, Burger, Ribal, Sylvester, van der Kwast, van Rhijn, Downes.

*Acquisition of data*: Beijert, Plass, Downes.

*Analysis and interpretation of data*: Beijert, van Rhijn, Downes.

*Drafting of the manuscript*: Beijert, van Rhijn, Downes.

*Critical revision of the manuscript for important intellectual content*: Beijert, Cheng, Liedberg, Plass, Williamson, Gontero, Ribal, Babjuk, Black, Kamat, Algaba, Berman, Hartmann, Masson-Lecomte, Roupret, Lopez-Beltran, Samaratunga, Shariat, Mostafid, Varma, Shen, Burger, Tsuzuki, Palou, Compérat, Sylvester, van der Kwast, van Rhijn, Downes.

*Statistical analysis*: Beijert, van Rhijn, Downes.

*Obtaining funding*: None.

*Administrative, technical, or material support*: None.

*Supervision*: van Rhijn, Downes.

*Other*: None.

  ***Financial disclosures:*** Irene J. Beijert certifies that all conflicts of interest, including specific financial interests and relationships and affiliations relevant to the subject matter or materials discussed in the manuscript (eg, employment/affiliation, grants or funding, consultancies, honoraria, stock ownership or options, expert testimony, royalties, or patents filed, received, or pending), are the following: None.

  ***Funding/Support and role of the sponsor:*** None.

  ***Acknowledgments:*** The present study was conducted under the auspices of the International Society of Urological Pathology (ISUP) and the European Association of Urology (EAU) - NMIBC Guidelines Panel. The survey was approved by the EAU Guidelines Office Board. We would like to thank all the experts and the ISUP and EAU members for taking the time to participate in the survey.

## References

[1] Sylvester R.J., Rodríguez O., Hernández V. (2021). European Association of Urology (EAU) Prognostic factor risk groups for non-muscle-invasive bladder cancer (NMIBC) incorporating the WHO 2004/2016 and WHO 1973 classification systems for grade: an update from the EAU NMIBC Guidelines Panel. Eur Urol.

[2] Sylvester R.J., Van der M.A., Lamm D.L. (2002). Intravesical bacillus Calmette-Guerin reduces the risk of progression in patients with superficial bladder cancer: a meta-analysis of the published results of randomized clinical trials. J Urol.

[3] Mostofi F.K.S., Sobin L.H., Torloni H., World Health Organization (1973).

[4] Epstein J.I., Amin M.B., Reuter V.R., Mostofi F.K. (1998). The World Health Organization/International Society of Urological Pathology consensus classification of urothelial (transitional cell) neoplasms of the urinary bladder. Bladder Consensus Conference Committee. Am J Surg Pathol.

[5] Busch C., Algaba F. (2002). The WHO/ISUP 1998 and WHO 1999 systems for malignancy grading of bladder cancer. Scientific foundation and translation to one another and previous systems. Virchows Arch.

[6] Bostwick D.G., Mikuz G. (2002). Urothelial papillary (exophytic) neoplasms. Virchows Arch.

[7] Humphrey P.A., Moch H., Cubilla A.L., Ulbright T.M., Reuter V.E. (2016). The 2016 WHO classification of tumours of the urinary system and male genital organs—part B: prostate and bladder tumours. Eur Urol.

[8] Netto G.J., Amin M.B., Berney D.M. (2022). The 2022 World Health Organization classification of tumors of the urinary system and male genital organs—part B: prostate and urinary tract tumors. Eur Urol.

[9] Soukup V., Čapoun O., Cohen D. (2017). Prognostic performance and reproducibility of the 1973 and 2004/2016 World Health Organization grading classification systems in non-muscle-invasive bladder cancer: a European Association of Urology non-muscle invasive bladder cancer guidelines panel systematic review. Eur Urol.

[10] van Rhijn B.W.G., Hentschel A.E., Bründl J. (2021). Prognostic value of the WHO1973 and WHO2004/2016 classification systems for grade in primary Ta/T1 non-muscle-invasive bladder cancer: a multicenter European Association of Urology Non-muscle-invasive Bladder Cancer Guidelines Panel study. Eur Urol Oncol.

[11] Hentschel A.E., van Rhijn B.W.G., Bründl J. (2020). Papillary urothelial neoplasm of low malignant potential (PUN-LMP): still a meaningful histo-pathological grade category for Ta, noninvasive bladder tumors in 2019?. Urol Oncol.

[12] van der Kwast T., Liedberg F., Black P.C. (2022). International Society of Urological Pathology expert opinion on grading of urothelial carcinoma. Eur Urol Focus.

[13] Bosschieter J., Hentschel A., Savci-Heijink C.D. (2018). Reproducibility and prognostic performance of the 1973 and 2004 World Health Organization classifications for grade in non-muscle-invasive bladder cancer: a multicenter study in 328 bladder tumors. Clin Genitourin Cancer.

[14] Babjuk M., Burger M., Capoun O. (2022). European Association of Urology guidelines on non-muscle-invasive bladder cancer (Ta, T1, and carcinoma in situ). Eur Urol.

[15] Chang S.S., Boorjian S.A., Chou R. (2016). Diagnosis and treatment of non-muscle invasive bladder cancer: AUA/SUO guideline. J Urol.

[16] Bobjer J., Hagberg O., Aljabery F. (2022). Bladder cancer recurrence in papillary urothelial neoplasm of low malignant potential (PUNLMP) compared to G1 WHO 1999: a population-based study. Scand J Urol.

[17] Downes M.R., Lajkosz K., Kuk C., Gao B., Kulkarni G.S., van der Kwast T.H. (2022). The impact of grading scheme on non-muscle invasive bladder cancer progression: potential utility of hybrid grading schemes. Pathology.

