# Supplementary material for: International Opinions on Grading of Urothelial Carcinoma: A Survey Among European Association of Urology and International Society of Urological Pathology Members
Source: Eur Urol Open Sci. 2023 May 10;52:154–65. doi: 10.1016/j.euros.2023.03.019 (PMC10240524; doi:10.1016/j.euros.2023.03.019)
Supplement: Supplementary Data 1 [file mmc1.docx]

**Supplementary material**

**Survey questions EAU-ISUP Bladder cancer grading survey 2021:**

1. Your main profession is:
   1. Urologist (- in training)
   2. Pathologist (-in training)
   3. Scientist/PhD
   4. Other, please specify
2. What is your main working environment?
   1. Community practice hospital
   2. Academic center / Medical school affiliated hospital
   3. Commercial third party hospital
   4. Other, please specify
3. How many cases of PUNLMP did you encounter in 2020
   1. Never
   2. Rarely, only once or twice
   3. Infrequent, 3-9
   4. Commonly, > 10
4. How many cases of non-invasive (Ta) urothelial carcinoma, excluding PUNLMP did you see in 2020
   1. 1-9
   2. 10-19
   3. 20-50
   4. > 50
5. Which grading system do you use when reporting a papillary non-invasive urothelial carcinoma
   1. WHO 1973 grading system
   2. WHO 2004 grading system
   3. Reporting both WHO 1973 and WHO 2004 grading system
   4. Other: please specify
6. Do you think treatment /management of PUNLMP should be different from Ta low grade urothelial carcinoma
   1. Yes
   2. No
   3. Not sure
   4. Other
7. Do you prefer for bladder cancer grading
   1. A 2-tier grading system
   2. A 3-tier grading system
   3. A 4-tier grading system
   4. No opinion
8. With regard to WHO 1973 grade 3 do you think (check your most favorite option)
   1. Its distinction from WHO 2004 high grade would influence clinical decisions in **Ta** tumours
   2. Its distinction from WHO 2004 high grade would influence clinical decisions in **Ta and T1** tumours
   3. Its distinction from WHO 2004 high grade does influence clinical decisions in **T1** tumours only
   4. Its distinction from WHO 2004 high grade would **not** influence clinical decisions in **Ta** or **T1** tumours
   5. No opinion
9. Would you consider reverting back to the WHO 1973 grading system
   1. Never
   2. Yes, as it is
   3. Only if grading criteria are more detailed
   4. Maybe, if further modifications were made
   5. Not applicable because I am still using WHO 1973
10. Do you think that a future grading system should be
    1. Unchanged, that is WHO 2004 classification: PUNLMP, low and high grade
    2. WHO 2004 classification minus PUNLMP, that is a 2-tier grading (Low and high grade)
    3. The previous WHO 1973 grading, that is 3-tier (grade 1, 2 and 3)
    4. The 4-tier WHO 1999 grading minus PUNLMP, that is splitting up WHO 2004 low grade into WHO 1973 G1 and G2 and splitting up WHO 2004 high grade into WHO 1973 G2 and G3.
    5. A hybrid 3-tier grading system, that is WHO 2004 low grade and WHO 2004 high grade split up into WHO 1973 G2 and G3
